# Supplementary material for: fastlin: an ultra-fast program for Mycobacterium tuberculosis complex lineage typing
Source: Bioinformatics. 2023 Oct 23;39(11):btad648. doi: 10.1093/bioinformatics/btad648 (PMC10627351; doi:10.1093/bioinformatics/btad648)
Supplement: btad648_Supplementary_Data [file btad648_supplementary_data.zip › Supplementary_material_2.pdf]

Supplementary material 2

This study uses high-quality MTB genome assemblies downloaded from the Refseq NCBI database and infers their lineage as follows:

- 150bp single-end Illumina reads representing 60x coverage are simulated using Art-Illumina (Huang et al. 2012)
- the simulated reads are then typed using TB-profiler v4.4.2

The following table indicates the number of false positive k-mer barcodes (i.e. barcodes that should not be detected given the lineages of these genome assemblies) obtained at different k-mer sizes. K-mer sizes ranging from 61 to 99 are not shown (0 false positives in all cases). The false positive found with all three genomes at k-mer sizes ranging from 19 to 47 is always the same one: a G at position 1882572 (specific to the lineage 4.9.1).

Kmer size	NC_000962.3 (lineage 4.9)	NZ_CP041872.1 (lineage 2.2.1)	NZ_CP041871.1 (lineage 3)
11	871	860	875
13	359	353	363
15	62	63	62
17	14	14	14
19	1	1	1
21	1	1	1
23	1	1	1
25	1	1	1
27	1	1	1
29	1	1	1
31	1	1	1
33	1	1	1
35	1	1	1
37	1	1	1
39	1	1	1
41	1	1	1
43	1	1	1
45	1	1	1
47	1	1	1
49	0	0	0
51	0	0	0
53	0	0	0
55	0	0	0
57	0	0	0
59	0	0	0

Huang, W., L. Li, J. R. Myers, and G. T. Marth. 2012. 'ART: a next-generation sequencing read simulator', *Bioinformatics*, 28: 593-4.
