# Supplementary material for: fastlin: an ultra-fast program for Mycobacterium tuberculosis complex lineage typing
Source: Bioinformatics. 2023 Oct 23;39(11):btad648. doi: 10.1093/bioinformatics/btad648 (PMC10627351; doi:10.1093/bioinformatics/btad648)
Supplement: btad648_Supplementary_Data [file btad648_supplementary_data.zip › Supplementary_material_3.pdf]

Supplementary materials 3

This file presents all 68 discrepancies between the lineage predictions made by TB-profiler and fastlin, and the 2 samples for which both TB-profiler and fastlin fail to infer any lineage.

Both TB-profiler and fastlin infer mixed samples but with different lineages (n=7)

SRA ID	TB-profiler lineage	fastlin lineage	fastlin log barcodes
SRR11972381	4, 1.1.3	4.8 (9), 1.1.3 (7)	1 (5, 7, 9, 7, 12, 5, 5, 5), 1.1 (6, 4, 6, 10, 12, 5, 7, 12), 1.1.3 (10, 7, 6, 12, 11, 7, 9, 4, 4), 4 (17, 10, 21, 8, 4, 16, 6, 20, 6, 15), 4.8 (8, 6, 11, 27, 11, 13, 9, 10, 8, 6)
SRR12199442	2, 1.2.2.2	1.2.2.2 (7), 1.2.1.2.1 (9)	1 (17, 56, 21, 11, 10, 12, 4, 15, 16, 14), 1.1 (6), 1.1.3 (8), 1.2.1 (7, 10, 35, 14, 8, 11, 6, 8, 12), 1.2.1.2 (7, 6, 5, 14, 11, 10, 6, 69), 1.2.1.2.1 (17, 13, 5, 7, 7, 4, 21, 8, 10, 14), 1.2.2 (4, 5, 5, 74, 7), 1.2.2.2 (17, 7, 18, 6, 5), 2 (4), 2.2 (4, 8), 2.2.1 (34), 3.1.1 (4)
SRR19355499	4.2.2.2, 3.1.1, 2, 1	3.1.1 (6), 2.2.1 (6)	1 (5), 1.1 (7), 2 (6, 4, 4, 7, 12, 9, 11, 11, 4, 4), 2.2 (4, 11, 4, 4, 10, 7, 13, 10), 2.2.1 (10, 5, 4, 6, 6, 4, 11), 3 (4, 7, 5), 3.1.1 (6, 4, 7, 9), 4.2 (4), 4.2.2 (4), 4.2.2.2 (4)
SRR19428521	2.2.1, 1.1.2	3.1.1 (11), 2.2 (5)	2 (4), 2.2 (4, 6, 7, 4), 3 (5, 8, 8, 8, 5, 7, 15, 14), 3.1.1 (12, 9, 6, 12, 14, 10), 4.2 (4)
SRR998662	6.3.1, 4.1.3	4.1 (4), 4.6.2.2 (4), 6.3 (4)	4 (5, 5, 12, 4, 7, 7, 11, 19, 7, 15), 4.1 (7, 4, 4, 4), 4.1.3 (4, 5), 4.6 (8, 7, 7, 4), 4.6.2 (6, 7, 7, 5, 7, 6), 4.6.2.2 (5, 4, 4, 4, 5, 4), 6 (4, 8), 6.3 (4, 4, 4, 5, 7), 6.3.1 (4, 4)

TB-profiler inferred mixed samples but fastlin did not (n= 8)

SRA ID	TB-profiler lineage	fastlin lineage	fastlin log barcodes
SRR11972253	2.2.1.1, 1.1.3	1.1.3 (12)	1 (6, 9, 11, 4, 8, 8, 9), 1.1 (9, 8, 4, 9, 10, 7, 4, 4, 4), 1.1.3 (19, 5, 18, 4, 8, 13, 5, 12, 22), 2 (4), 2.2.1 (4), 2.2.1.1 (4)
ERR4797054	6.3, 2.1	2.2.1.2 (28)	1.1.2 (74), 1.2.1.2.1 (47), 2 (34, 27, 22, 14, 22, 18, 22, 29, 28, 26), 2.2 (21, 38, 22, 26, 29, 19, 16, 25, 15, 25), 2.2.1 (14, 35, 37, 14, 7, 24, 20, 16, 39), 2.2.1.2 (36, 25, 31, 25)
SRR1181390	4.9, 2.2	2.2.1 (5)	2 (7, 5), 2.2 (4, 6, 4), 2.2.1 (5, 8, 5, 5)
SRR1196677	2.2, La3	2.2.1 (6)	1.1.2 (4), 1.2.1.2.1 (5), 2 (9, 5, 4, 4, 4, 5, 7), 2.2 (4, 4, 4, 5, 8), 2.2.1 (5, 7, 5, 6, 9)
SRR20969677	6.3, La3	2.1 (51)	1.1.2 (193), 1.2.1.2.1 (185), 2 (49, 40, 47, 52, 55, 37, 35, 49, 94, 83), 2.1 (33, 49, 43, 73, 60, 95, 53, 91, 48, 38)
SRR023458	4.9, 4.1.2.1	4.1.2.1 (221)	1.1 (6), 1.2.1 (4), 1.2.1.2.1 (5), 3.1.1 (8), 4 (342, 321, 442, 601, 401, 12, 299, 341, 392, 107), 4.1 (498, 582, 269, 572, 247, 438, 361, 528, 180, 52), 4.1.1.1 (5), 4.1.1.2 (4), 4.1.1.3 (5), 4.1.1.3.1 (4), 4.1.2 (142, 111, 277, 539, 271, 368, 136, 128, 392, 318), 4.1.2.1 (430, 161, 209, 216, 111, 315, 684, 467, 227, 168), 4.1.4 (4), 4.2.2.2 (5), 4.3.2 (4), 4.3.4.2.1 (4), 4.6 (29), 4.7 (6), 4.8 (4), 4.9 (4), 5.1.2 (4), 6.1.3 (4), La1.3 (7), La1.8.2 (4, 4)
ERR3283010	4.9;2.1, La1.8.1, La1.7.1	La1.8.1 (4)	8 (5), La1 (4, 5, 5), La1.8 (4, 5, 4, 4), La1.8.1 (4, 5, 4)
ERR4798262	La2, La1.8.2, La1.2.BCG	1.1.2 (16)	1 (12, 19, 14, 18, 11, 14, 16, 24, 21, 20), 1.1 (13, 23, 20, 17, 23, 6, 11, 14, 23, 16), 1.1.2 (12, 17, 24, 16, 7, 16, 17, 18, 13, 19)

fastlin inferred mixed samples but TB-profiler did not (n= 36)

SRA ID	TB-profiler lineage	fastlin lineage	fastlin log barcodes
SRR19428508	1.1.2	4.1 (6), 1.1.2 (59)	1 (48, 71, 55, 23, 55, 89, 66, 61, 99, 89), 1.1 (82, 84, 106, 89, 90, 59, 89, 73, 79, 94), 1.1.2 (29, 79, 62, 30, 59, 58, 59, 101, 95, 57), 4 (4, 4), 4.1 (5, 6, 7), 4.1.1 (6, 5), 4.1.1.1 (4, 5)
ERR176819	1.1.3.2	1.1.3.2 (72), 4.3.4.2 (4)	1 (83, 72, 73, 95, 73, 66, 72, 70, 67, 83), 1.1 (99, 82, 77, 86, 58, 39, 82, 73, 82, 71), 1.1.3 (74, 50, 77, 68, 76, 79, 70, 70, 68, 79), 1.1.3.2 (58, 63, 66, 75, 79, 69, 75, 84), 2.2.1 (4), 4 (6), 4.2.2 (4), 4.3.4 (5, 5, 5), 4.3.4.2 (4, 4, 7), 4.3.4.2.1 (4)
ERR221621	1.1.3.2	1.2.2.2 (8), 1.1.3.2 (131)	1 (148, 110, 148, 122, 135, 127, 148, 124, 129, 120), 1.1 (133, 160, 113, 125, 157, 120, 159, 125, 108, 100), 1.1.3 (134, 108, 111, 139, 147, 213, 114, 145, 125, 138),

			1.1.3.2 (113, 148, 135, 143, 127, 101, 135, 104), 1.2.2 (6, 18, 9, 6, 7, 8, 8, 11, 8, 9), 1.2.2.2 (8, 5, 8, 7, 6, 8, 11, 6, 12)
ERR221666	1.1.3.2	3.1.1 (9), 1.1.3.2 (108)	1 (125, 99, 118, 100, 103, 105, 124, 103, 93, 105), 1.1 (106, 123, 111, 129, 131, 92, 105, 102, 113, 122), 1.1.3 (102, 103, 132, 124, 116, 117, 123, 135, 119, 107), 1.1.3.2 (111, 82, 101, 106, 119, 115, 135, 98), 3 (11, 11, 6, 6, 4, 4, 10, 4, 6), 3.1.1 (12, 9, 9, 11, 9, 4, 6, 4)
SRR10809195	1.2.1	4.4.1.2 (5), 1.2.1 (104)	1 (97, 145, 92, 113, 139, 51, 105, 109, 139, 120), 1.2.1 (125, 99, 96, 82, 109, 80, 123, 127, 129, 41), 4 (7, 4, 4, 4), 4.4 (7, 4), 4.4.1 (4, 5, 5, 4, 6), 4.4.1.2 (5, 5, 6, 4, 4)
SRR1011492	1.2.2.1	1.2.2.1 (93), 4 (4)	1 (43, 104, 113, 12, 93, 120, 129, 85, 145, 42), 1.1.2 (4), 1.2.2 (145, 58, 116, 146, 54, 128, 120, 106, 83, 89), 1.2.2.1 (34, 41, 29, 32, 144, 124, 125, 91, 112, 95), 2.2 (4, 4), 4 (4, 5, 4), 4.3 (4, 4), 4.3.2 (5), 4.3.2.1 (5, 5)
SRR1013592	2	2 (106), 4 (4)	2 (159, 113, 73, 92, 119, 116, 107, 101, 105, 21), 4 (5, 4, 4)
ERR4799973	3.1	3 (76), 1 (5)	1 (5, 5, 7), 1.1 (4), 2 (4, 5), 2.2 (4), 2.2.1 (6, 6), 3 (53, 76, 63, 93, 94, 68, 79, 86, 76), 3.1 (78)
ERR688048	3	4.3.4.2 (6), 3 (384)	1.2.1 (4), 3 (384, 416, 451, 395, 353, 219, 452, 360, 361), 4 (4, 4, 4, 6, 5), 4.1.4 (4), 4.3 (4, 4, 6, 4), 4.3.4 (4, 12, 4), 4.3.4.2 (6, 8, 5, 7, 6), 4.6 (4), 5.1.2 (6), 8 (4)
SRR4035524	3	3 (257), 4.1.2 (6)	3 (205, 277, 308, 271, 161, 99, 257, 304, 236), 4 (4, 4), 4.1.2 (6, 4, 10), 4.2 (4)
ERR400377	4.1.2	4.1.2 (71), 4.3.4.2 (5)	4 (89, 71, 70, 75, 50, 110, 70, 108, 26, 95), 4.1 (148, 96, 83, 104, 74, 76, 77, 58, 105, 72), 4.1.2 (72, 105, 70, 71, 55, 70, 81, 103, 65, 113), 4.3.4.2 (7, 5, 4), 4.3.4.2.1 (12)
ERR2514497	4.1.3	4.1.3 (98), 1.1.2 (5)	1 (4), 1.1 (6, 8, 4), 1.1.2 (4, 7, 4, 7), 4 (108, 116, 92, 109, 110, 88, 119, 67, 131, 71), 4.1 (138, 114, 131, 120, 103, 106, 102, 78, 132, 97), 4.1.3 (108, 106, 83, 108, 115, 91, 75, 90, 141, 75)
SRR998798	4.1.3	4.6.2.2 (8), 4.1.3 (64)	4 (69, 64, 62, 72, 82, 68, 62, 37, 84, 56), 4.1 (90, 78, 62, 79, 71, 74, 48, 53, 93, 70), 4.1.3 (67, 79, 44, 54, 83, 53, 62, 74, 61, 74), 4.6 (6, 6, 7), 4.6.2 (4, 5, 5, 6, 9, 7, 8, 5), 4.6.2.2 (8, 10, 4)
ERR234686	4.2.1	4.2.1 (112), 2.2.1 (4)	2 (7, 4, 4, 6, 5), 2.2.1 (4, 4, 5), 4 (105, 118, 102, 110, 94, 139, 106, 123, 84, 122), 4.2 (113, 87, 104, 113, 114, 95, 87, 108, 98, 116), 4.2.1 (115, 96, 85, 126, 109, 121, 119, 102, 120, 106)
SRR20969577	4.2.2	4.2.2 (247), 2.2.1.1 (19)	2 (21, 15, 30, 7, 20, 21, 14, 16, 18, 20), 2.2 (16, 16, 19, 22, 15, 27, 30, 7, 19, 25), 2.2.1 (27, 21, 11, 14, 13, 13, 15, 14, 22), 2.2.1.1 (16, 18, 21, 31, 25, 15), 4 (249, 256, 245, 240, 277, 225, 257, 256, 267, 221), 4.2 (242, 223, 267, 244, 285, 149, 287, 230, 259, 200), 4.2.2 (210, 258, 217, 254, 259, 191, 250, 244)
SRR5818596	4.2	3 (4), 4.2 (33)	3 (4, 9, 4), 4 (45, 33, 37, 32, 46, 19, 48, 28, 23, 36), 4.2 (45, 35, 65, 73, 35, 12, 31, 31, 26, 25), 4.6 (32)
ERR8699189	4.3.2.1	2.2 (5), 4.3.2.1 (137), 3 (10)	2.2 (6, 5, 5), 3 (12, 6, 11, 10, 13, 7, 6, 19, 9), 4 (171, 147, 174, 162, 119, 149, 116, 126, 126, 136), 4.3 (167, 159, 140, 126, 139, 96, 133, 166, 140), 4.3.2 (140, 145, 145, 148, 168, 105, 143, 139, 170, 168), 4.3.2.1 (126, 143, 134, 176, 140, 118, 156, 128, 118, 169)
ERR212171	4.3.4.2.1	4.3.4.2.1 (116), 3 (5)	3 (5, 4, 5), 3.1.1 (6), 4 (153, 150, 117, 70, 164, 162, 117, 116, 89, 146), 4.3 (104, 145, 156, 112, 96, 110, 74, 155, 83), 4.3.2 (6), 4.3.4 (83, 89, 135, 144, 142, 92, 111), 4.3.4.2 (67, 97, 159, 151, 69, 162, 116, 72, 150, 129), 4.3.4.2.1 (85, 119, 140, 115, 126, 68, 118, 111)
SRR847802	4.4.1.1.1	4.9 (5), 4.1.2.1 (5), 4.4.1.1.1 (818), 2.2.1 (4)	1.1 (5), 1.2.1.2 (4), 1.2.1.2.1 (13), 1.2.2.1 (8), 1.2.2.2 (5), 2 (6), 2.1 (4), 2.2.1 (4, 8, 4), 2.2.1.1 (7), 3 (4), 3.1.1 (5, 7), 3.1.2.2 (4), 3.1.3 (4), 4 (644, 864, 721, 837, 825, 740, 774, 731, 820, 791), 4.1 (4, 4, 5, 4, 4, 5, 7), 4.1.1.1 (5, 4), 4.1.1.2 (13), 4.1.2 (6, 4, 6, 4), 4.1.2.1 (4, 6, 5, 4, 9), 4.1.2.1.1 (4), 4.2 (4), 4.2.1.1 (4), 4.3.4 (4), 4.4 (844, 854, 767, 759, 883, 850, 851, 817, 819, 655), 4.4.1 (729, 789, 890, 823, 716, 845, 931, 772, 773, 769), 4.4.1.1 (915, 934, 807, 783, 958, 826, 798, 640, 859, 840), 4.4.1.1.1 (818, 985, 504, 821, 877, 759, 749, 911, 733), 4.4.2 (4), 4.6 (5), 4.6.1.2 (6), 4.6.3 (4), 4.6.5 (4), 4.7 (4), 4.8.1 (4), 4.9 (5, 5, 4), 4.9.1 (4, 4), 5 (8), 5.1.2 (23), 5.1.3 (5), 5.1.4 (5), 5.2 (6), 5.3 (4), 6.1.3 (4, 4), 7 (4, 5), 8 (11), 9 (5), La1.2.BCG (4), La1.3 (6, 7), La1.4 (4), La1.7.X-unk4 (8), La1.8 (4), La3 (9)
SRR4037763	4.4.1.1	2.2.1 (5), 4.4.1.1 (85)	2 (4, 4, 8, 7, 5, 5), 2.2 (5, 13, 10, 5, 5, 7, 8, 7), 2.2.1 (9, 5, 5, 9, 6, 4), 4 (69, 56, 80, 69, 71, 72, 95, 77, 92, 78), 4.4 (85, 129, 61, 43, 112, 76, 69, 49, 133, 68), 4.4.1 (60, 73, 110, 78, 68, 87, 35, 30, 53, 71), 4.4.1.1 (52, 76, 125, 130, 91, 87, 52, 90, 75, 83), 8 (4)
SRR15368744	4.4.2	2.2 (6), 4.4.2 (250)	2 (9, 7, 4, 4), 2.2 (6, 4, 7), 2.2.1 (4, 5), 2.2.1.1 (4), 4 (299, 223, 318, 275, 268, 225, 318, 309, 211, 275), 4.4 (268, 305, 298, 228, 227, 334, 317, 333, 295, 223), 4.4.2 (222, 272, 229, 307, 193, 284), 6 (4)
SRR8651637	4.4.2	2.2 (4), 4.4.2 (321)	2.2 (4, 4, 4), 2.2.1 (4), 4 (252, 317, 365, 235, 197, 283, 329, 233, 353, 301), 4.4 (319, 130, 387, 303, 166, 451, 299, 288, 335, 365), 4.4.2 (316, 305, 326, 383, 350, 205)
ERR1199115	4.6.1.2	4.3.4.2.1 (7), 4.6.1.2 (123)	4 (87, 122, 120, 31, 168, 160, 136, 94, 127, 115), 4.3 (4, 5, 7, 4, 7, 9, 7, 6), 4.3.4 (4, 6, 5, 5, 4), 4.3.4.2 (9, 9, 8, 6, 5, 5, 4), 4.3.4.2.1 (8, 5, 7, 8, 5, 8, 4), 4.6 (147, 118, 86, 123, 55, 114, 133), 4.6.1 (102, 109, 150, 78, 282, 93, 148, 116, 81, 121), 4.6.1.2 (130, 125, 121, 121, 164, 102, 122, 120, 130, 170)
ERR2707224	4.6.1.2	4.3.4.2.1 (7), 4.6.1.2 (123)	4 (87, 122, 120, 31, 168, 160, 136, 94, 127, 115), 4.3 (4, 5, 7, 4, 7, 9, 7, 6), 4.3.4 (4, 6, 5, 5, 4), 4.3.4.2 (9, 9, 8, 6, 5, 5, 4), 4.3.4.2.1 (8, 5, 7, 8, 5, 8, 4), 4.6 (147, 118, 86, 123, 55, 114, 133), 4.6.1 (102, 109, 150, 78, 282, 93, 148, 116, 81, 121), 4.6.1.2 (130, 125, 121, 121, 164, 102, 122, 120, 130, 170)
SRR18213698	4.6	1.1.3 (4), 4.6 (66)	1 (4, 6), 1.1 (4, 4), 1.1.3 (4, 4, 5), 4 (66, 40, 101, 45, 69, 39, 87, 66, 73, 82), 4.6 (81, 46, 67, 66, 65, 78, 64)
SRR2101048	4.6	1.1.3 (4), 4.6 (66)	1 (4, 6), 1.1 (4, 4), 1.1.3 (4, 4, 5), 4 (66, 40, 101, 45, 69, 39, 87, 66, 73, 82), 4.6 (81, 46, 67, 66, 65, 78, 64)

ERR266580	4.8.2	4.8.2 (65), 4.6 (6)	4 (59, 64, 33, 96, 43, 74, 68, 52, 74, 68), 4.6 (5, 6, 6), 4.6.3 (5), 4.8 (56, 63, 76, 59, 60, 23, 89, 69, 66, 52), 4.8.2 (55, 96, 66, 78, 70, 74, 54, 51, 33, 65), 5.1.2 (4)
ERR245719	4.9.1	4.3.4.2.1 (7), 4.9.1 (159)	1.2.1.2.1 (5), 4 (155, 164, 268, 193, 163, 151, 196, 160, 253, 118), 4.1.4 (6), 4.2.2 (4), 4.3 (4, 4, 4, 7, 5, 6, 8, 7), 4.3.4 (8, 13, 4, 7), 4.3.4.2 (4, 7, 4, 5, 6, 4, 6, 5, 7), 4.3.4.2.1 (7, 7, 4, 7, 4), 4.6 (7), 4.9 (117, 221, 174, 193, 152, 186, 188, 201, 231, 181), 4.9.1 (165, 125, 158, 201, 159, 239, 227, 111, 130), La1.7.X-unk4 (6)
ERR4799905	4.9	4.9 (110), 2 (5)	2 (4, 5, 8), 2.2 (5), 2.2.1 (7, 4), 3 (6), 4 (116, 84, 100, 92, 99, 90, 82, 100, 105, 108), 4.9 (86, 125, 125, 75, 101, 83, 119, 120, 123, 58)
ERR3801606	6.1.3	6.1.3 (228), 4.1.2.1 (6)	4 (6, 12, 6, 4, 6, 8, 5), 4.1 (12, 7, 7, 4, 4, 7, 5), 4.1.2 (7, 5, 5, 5, 6), 4.1.2.1 (4, 4, 8, 9, 5, 10), 6 (236, 111, 172, 209, 196, 170, 179, 284, 181, 175), 6.1.3 (262, 242, 215, 191), La1.3 (5)
ERR9787253	6.1.3	6.1.3 (228), 4.1.2.1 (6)	4 (6, 12, 6, 4, 6, 8, 5), 4.1 (12, 7, 7, 4, 4, 7, 5), 4.1.2 (7, 5, 5, 5, 6), 4.1.2.1 (4, 4, 8, 9, 5, 10), 6 (236, 111, 172, 209, 196, 170, 179, 284, 181, 175), 6.1.3 (262, 242, 215, 191), La1.3 (5)
SRR998606	6.2.1	6.2.1 (93), 6.3.1 (5)	6 (103, 109, 58, 76, 103, 67, 73, 111, 116, 102), 6.2 (86, 104, 102, 78, 85, 112, 69, 94, 91), 6.2.1 (104, 83, 104, 69), 6.3 (4, 5, 9, 4, 4, 8), 6.3.1 (5, 9, 4, 4, 7, 6)
SRR998607	6.2.1	6.3 (4), 6.2.1 (87)	6 (92, 71, 92, 78, 68, 90, 84, 129, 82, 87), 6.2 (77, 88, 101, 55, 66, 113, 74, 106, 70), 6.2.1 (125, 87, 88, 53), 6.3 (6, 4, 5, 4, 4, 4, 9), 6.3.1 (4, 7)
ERR3170439	6.3.3	6.3.3 (83), 4 (4)	4 (4, 4, 4, 4), 4.6 (4), 4.6.2 (4), 4.6.2.2 (4), 6 (113, 100, 65, 99, 105, 113, 91, 113, 119, 109), 6.3 (107, 78, 89, 106, 91, 93, 79, 139, 85), 6.3.3 (83, 128, 83, 89, 81, 105, 77, 125, 84, 78)
SRR998576	6.3.3	6.3.3 (62), 6.3.1 (6)	6 (84, 58, 61, 72, 74, 57, 76, 72, 61, 88), 6.3 (56, 64, 82, 76, 67, 101, 67, 64, 57), 6.3.1 (6, 4, 6), 6.3.3 (42, 62, 57, 54, 64, 55, 74, 63, 68, 84)
ERR234682	La3	4 (5), La3 (97)	4 (5, 5, 7, 5, 4, 4, 4), 4.8 (6, 4), La3 (112, 101, 91, 97, 97)

TB-profiler failed to infer any lineage (n=8)

SRA ID	TB-profiler lineage	fastlin lineage	fastlin log barcodes
ERR4819092		1.2.1.2.1	1 (7, 4, 8, 5), 1.2.1 (11, 7, 5, 10), 1.2.1.2 (4, 5, 6, 5, 5), 1.2.1.2.1 (10, 4, 5, 6, 6, 9, 5, 5)
SRR21735256		1.2.2.2	1 (4, 10, 13, 31, 10, 5), 1.2.2 (13, 8, 4, 45, 8, 7), 1.2.2.2 (7, 16, 11, 33), 4.1.2 (12)
SRR12199408		4 (6), 1.2.2.2 (23)	1 (24, 23, 36, 46, 35, 30, 17, 12, 11, 25), 1.1.1 (17), 1.2.2 (18, 36, 29, 10, 22, 35, 35, 18, 20, 10), 1.2.2.2 (28, 37, 15, 23, 37, 28, 18, 23, 23), 2.2 (5, 5), 4 (10, 6, 5), 4.3 (4)
ERR6358755		4.6.1.1	1.1.2 (58), 1.2.1.2.1 (68), 2.1 (4), 4 (91, 47, 66, 65, 10, 112, 98, 51, 40, 69), 4.3.4 (4), 4.3.4.2 (6), 4.3.4.2.1 (4, 4), 4.4 (82), 4.6 (75, 50, 59, 55, 35, 66, 57), 4.6.1 (61, 74, 98, 38, 118, 54, 75, 79, 56, 68), 4.6.1.1 (60, 67, 50, 103, 64, 75, 63, 59, 76, 46)
SRR5341203		3	3 (7, 9, 18, 17, 16, 6, 4, 17, 12)
ERR4187744		La1.8.1	La1 (4, 6, 9), La1.8 (8, 7), La1.8.1 (13, 7, 5, 4)
ERR3468577		La1.8.1	La1 (4), La1.8 (8, 5, 4), La1.8.1 (5, 6, 9, 6)
ERR4769482		La1	La1 (5, 4, 4), La1.3 (5, 4)

fastlin failed to infer any lineage (n=1)

SRA ID	TB-profiler lineage	fastlin lineage	fastlin log barcodes
ERR025887	4.3.4.2, 1.2.1.2.1		1 (4), 1.2.1 (6), 1.2.1.2 (4), 1.2.1.2.1 (4, 4), 4.3.4 (5)

Both TB-profiler and fastlin failed to infer any lineage (n=2)

SRA ID	TB-profiler lineage	fastlin lineage	fastlin log barcodes
ERR2747660			La1 (6, 6), La1.8 (6, 5), La1.8.1 (10)
ERR4769545			La1 (4)

fastlin provided a more precise lineage prediction (n=6)
Lineages identified by phylogenetic analyses (see below) are highlighted in bold green.

SRA ID	TB-profiler lineage	fastlin lineage	fastlin log barcodes
SRR10305655	**2.2.1**	2.2.1.1	2 (244, 259, 264, 242, 225, 197, 198, 234, 245, 266), 2.2 (191, 250, 279, 187, 234, 207, 275, 256, 202, 220), 2.2.1 (224, 199, 270, 237, 203, 248, 239, 204, 269), 2.2.1.1 (9, 7, 5, 4, 7)
SRR1062937	4.6.1	**4.6.1.1**	4 (16, 22, 7, 6, 17, 9, 9, 9, 13), 4.6 (14, 9, 20, 15, 4, 5, 15), 4.6.1 (15, 7, 9, 11, 10, 9, 8, 9, 11, 10), 4.6.1.1 (17, 15, 20, 10, 8, 6, 7, 13)
ERR1213918	4.6	**4.6.2.2**	4 (10, 33, 16, 36, 37, 16, 29, 111, 29, 45), 4.6 (43, 62, 23, 46, 13, 40, 34), 4.6.2 (25, 15, 9, 41, 13, 48, 26, 93, 33, 41), 4.6.2.2 (7, 39, 57, 43, 37, 35, 8, 39, 20)
ERR9787030	6	**6.1.2**	6 (65, 105, 41, 60, 59, 82, 94, 96, 100, 100), 6.1.2 (60, 59, 120, 103, 81, 115)
ERR3468727	La1.8	**La1.8.1**	La1 (4, 5), La1.8 (5, 5, 4, 6), La1.8.1 (5, 4, 5)
SRR10993935	La1	**La1.6**	La1 (7, 5), La1.6 (5, 5, 5)

TB-profiler and fastlin lineage predictions totally disagree (n=2)
Lineages identified by phylogenetic analyses (see below) are highlighted in bold green.

SRA ID	TB-profiler lineage	fastlin lineage	fastlin log barcodes
ERR9787014	La2	**6.1.1**	4.1.1.1 (63), 6 (136, 86, 148, 115, 118, 90, 126, 104, 122, 155), 6.1.1 (122, 128, 118, 124, 149, 129, 150, 157, 146, 122)
ERR9787139	2.1	**6.3.1**	1.1.2 (73), 6 (119, 58, 60, 106, 101, 80, 104, 123, 123, 87), 6.3 (96, 84, 116, 55, 95, 161, 72, 120, 103), 6.3.1 (109, 92, 57, 82, 78, 102, 88, 130, 88, 91)

In order to investigate further the discrepancies observed between TB-profiler and fastlin lineage predictions, we performed phylogenetic analyses of samples belonging to lineages 2, 4.6, 6 and La1. These lineages include the 8 non-mixed samples for which TB-profiler and fastlin inferred different lineages.

For these analyses, we selected all SRA samples (out of the 3139 samples that served as the basis for our comparison) belonging to one of these four lineages using the following criteria: samples without detected mixture by either TB-profiler or fastlin, having a kmer coverage between 30x and 300x (kmer coverages were estimated by fastlin in this study).

The sequencing reads of each sample were mapped onto the H37Rv reference genome using BWA mem v0.7.17 (Li 2013). The BAM files were then processed to filter out alignments with quality scores below 30 using SAMtools v1.10 (Danecek *et al.* 2021). Finally, SNPs and indels were called using BCFtools v1.10.2 (Danecek *et al.* 2021) using a minimum base quality score of 30.

We built a SNP alignment for each of the four lineages by selecting SNPs as follows: SNPs had to have a minimum coverage of 5x and a within-sample frequency of at least 0.75, be supported by at least one forward read and one reverse read, SNPs having, and not be found within 10 bases from any other SNP or indel in the same sample. The alignments were then analysed using IQ-TREE v2.2.2.7 (Nguyen et al. 2015) under a GTR model. Maximum likelihood trees were finally rooted using the midpoint root method - I would have used a

root-to-tip regression, unless there was a compelling reason not to (although that one does need a timestamp to be known for all the extant samples, so maybe it is not realistic here).

The phylogenetic trees can be found in Supplementary data 1 (last spreadsheet). The following figures show the rooted trees with (i) samples with conflicting lineage predictions indicated by an arrow and their name, and (ii) sub-lineages highlighted by colour boxes (samples without a colour box belong to the coarser lineage (e.g., lineage 2 in the tree immediately below)).

Phylogenetic tree of samples belonging to Lineage 2:

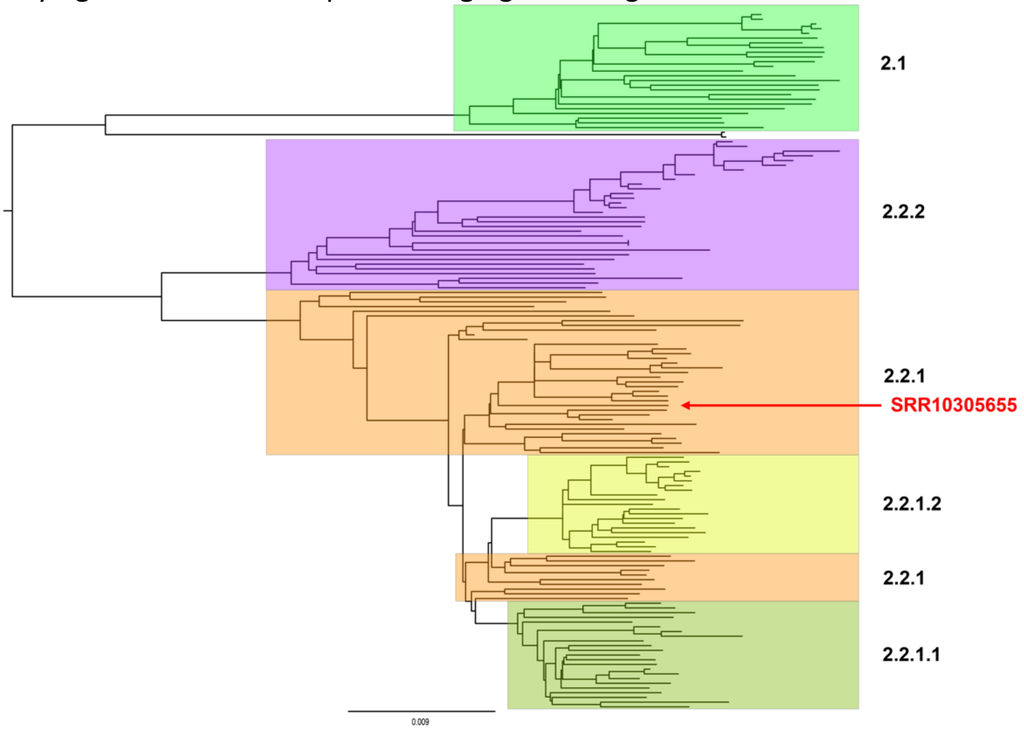

Phylogenetic tree of samples belonging to Lineage 4.6:

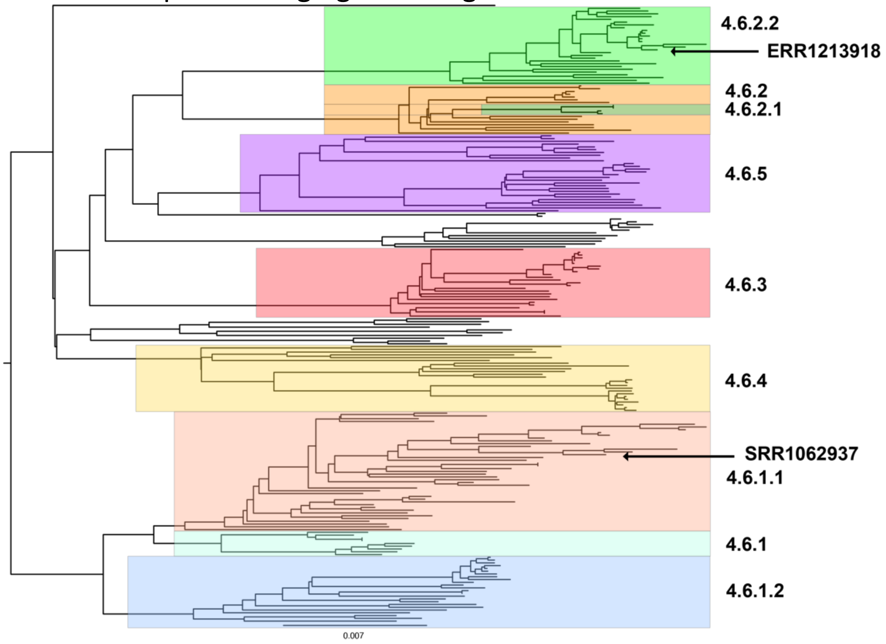

Phylogenetic tree of samples belonging to Lineage 6:

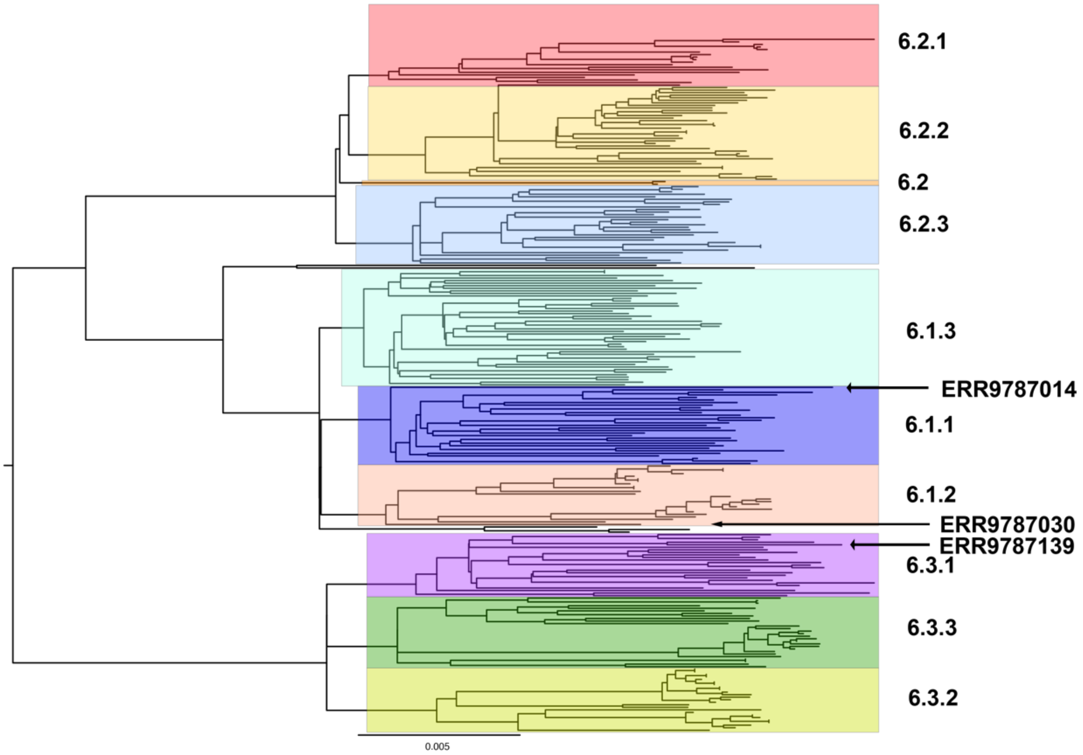

Phylogenetic tree of samples belonging to Lineage La.1 (La.1.7 sub-lineages not shown):

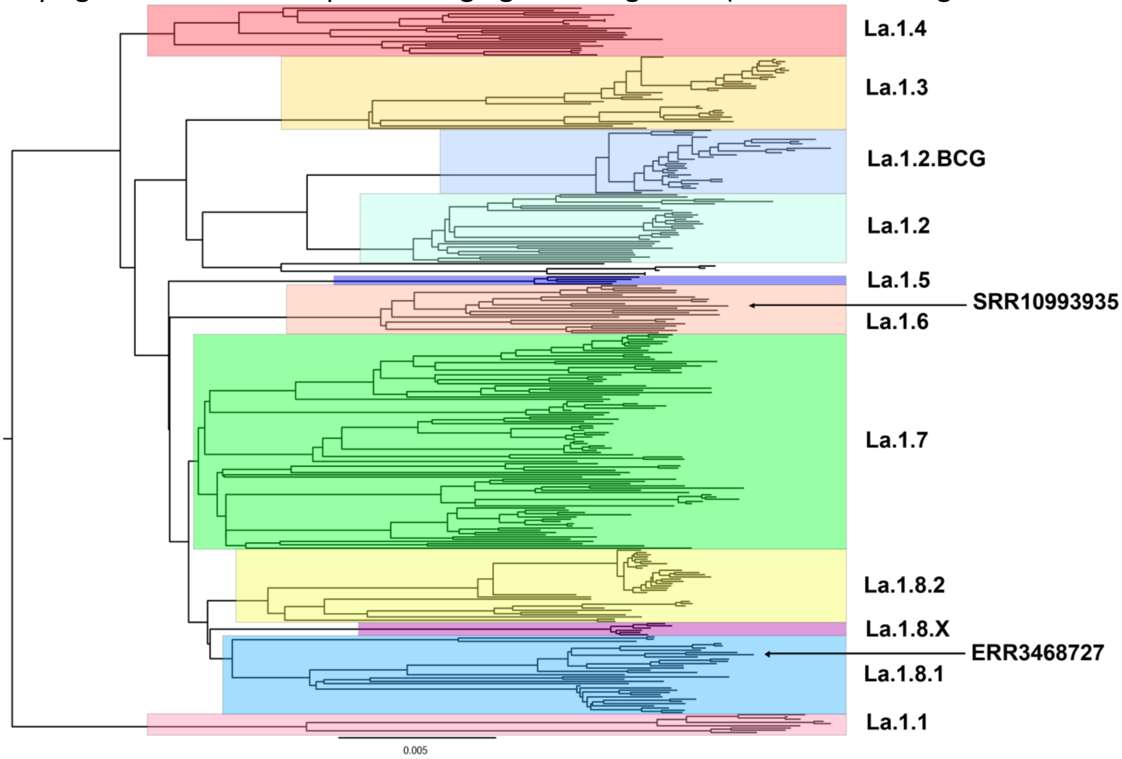

Li H. 2013. Aligning sequence reads, clone sequences and assembly contigs with BWA-MEM. arXiv:1303.3997v2.

Danecek P, Bonfield JK, Liddle J, Marshall J, Ohan V, Pollard MO, Whitwham A, Keane T, McCarthy SA, Davies RM, Li H. 2021. Twelve years of SAMtools and BCFtools. GigaScience.

Nguyen LT, Schmidt HA, von Haeseler A, and Minh BQ. 2015. IQ-TREE: A fast and effective stochastic algorithm for estimating maximum likelihood phylogenies. Mol. Biol. Evol., 32, 268-274.
